# Overweight and obesity predict better overall survival rates in cancer patients with distant metastases

**DOI:** 10.1002/cam4.634

**Published:** 2016-01-26

**Authors:** Ngan Ming Tsang, Ping Ching Pai, Chi Cheng Chuang, Wen Ching Chuang, Chen Kan Tseng, Kai Ping Chang, Tzu Chen Yen, Jen Der Lin, Joseph Tung Chieh Chang

**Affiliations:** ^1^ Department of Radiation OncologyChang Gung Memorial Hospital and University at Lin‐KouTaoyuanTaiwan; ^2^ School of Traditional Chinese MedicineChang Gung UniversityTaoyuanTaiwan; ^3^ Department of NeurosurgeryChang Gung Memorial Hospital and University at Lin‐KouTaoyuanTaiwan; ^4^ School of MedicineChang Gung UniversityTaoyuanTaiwan; ^5^ Departments of Otolaryngology‐ Head Neck SurgeryChang Gung Memorial Hospital and Chang Gung University at Lin‐KouTaoyuanTaiwan; ^6^ Departments of Nuclear Medicine and Molecular Imaging CenterChang Gung Memorial Hospital and University at Lin‐KouTaoyuanTaiwan; ^7^ Department of Internal MedicineChang Gung Memorial Hospital and University at Lin‐KouTaoyuanTaiwan

**Keywords:** BMI, body mass index, metastatic cancer, obesity, overall survival.

## Abstract

Recent studies conducted in patients with chronic diseases have reported an inverse association between body mass index (BMI) and mortality. However, the question as to whether BMI may predict prognosis in patients with metastatic cancer remains open. We therefore designed the current retrospective study to investigate the potential association between BMI and overall survival (OS) in patients with distant metastases (DM) and a favorable performance status. Between 2000 and 2012, a total of 4010 cancer patients with DM who required radiotherapy (RT) and had their BMI measured at the initiation of RT were identified. The relation between BMI and OS was examined by univariate and multivariable analysis. The median OS time was 3.23 months (range: 0.1–122.17) for underweight patients, 6.08 months (range: 0.03–149.46) for normal‐weight patients, 7.99 months (range: 0.07–158.01) for overweight patients, and 12.49 months (range, 0.2–164.1) for obese patients (log‐rank: *P *<* *0.001). Compared with normal‐weight patients, both obese (HR = 0.676; 95% *P *<* *0.001) and overweight individuals (HR = 0.84; *P *<* *0.001) had a reduced risk of all‐cause mortality in multivariable analysis. Conversely, underweight patients had a significantly higher risk of death from all causes (HR = 1.41; *P *<* *0.001). Overweight and obesity are independent predictors of better OS in metastatic patients with a good performance status. Increased BMI may play a role to identify metastatic patients with superior survival outcome and exhibit a potential to encourage aggressive management in those patients even with metastases.

## Introduction

Cancer remains a leading cause of death and a major public health concern worldwide. According to the U.S. Centers for Disease Control and Prevention report, approximately 12.7 million cancer cases are newly diagnosed and 7.6 million cancer deaths occur each year globally. Distant metastases (DM) are responsible of ~90% of all deaths caused by malignancies 1. The presence of DM portends a dismal prognosis in the vast majority of cancer patients and palliative radiotherapy for symptom relief remains the mainstay for their clinical management. Depending on cancer histology, performance status, and toxicity profile, some metastatic patients may be also treated with multiple courses of chemotherapy 2, 3, 4. Surgical removal of selected metastases is indicated only in highly specific circumstances 5, 6, 7. However, recent progresses in systemic chemotherapy‐ and molecular‐targeted therapy have significantly improved both survival and quality of life in cancer patients with DM. Moreover, recent diagnostic advances in functional imaging and molecular diagnostics have allowed earlier detection of isolated metastases. In this scenario, the use of aggressive systemic and local therapeutic approaches may be more suitable than palliation at least in selected patients 8. Although DM remain a major issue in cancer management, limited data are currently available on the selection of metastatic patients for treatment with curative intent.

Several recent studies conducted in patients with chronic diseases have reported an inverse association between body mass index (BMI) – defined as the weight in kilograms divided by the square of the height in meters – and mortality 9, 10, 11. This phenomenon, termed obesity paradox, has been initially described in patients with chronic renal failure undergoing hemodialysis 12. Recently, a large meta‐analysis examining nearly 100 studies conducted in more than 2.88 million adult patients confirmed that overweight (BMI = 25−30 kg/m^2^) is significantly associated with a lower risk of all‐cause mortality 13. However, overweight and obesity have been repeatedly associated with a higher risk of developing different types of cancer, including breast, colon, gallbladder, renal, endometrial, and esophageal malignancies 14, 15. Numerous studies have investigated the potential association of BMI with clinical outcomes in cancer patients. An increased BMI has been associated with worse outcomes in colorectal, breast, prostate, liver, pancreas, and esophageal malignancies. However, an inverse association between BMI and mortality has been reported in patients with lung cancer, renal cellar carcinoma, diffuse large B‐cell lymphoma, gastric cancer, and resected esophageal carcinoma 16, 17, 18, 19, 20, 21, 22. Unfortunately, the question as to whether BMI may predict prognosis in patients with metastatic cancer remains open. We therefore designed the current retrospective study to investigate the potential association between BMI and overall survival (OS) in patients with DM and a favorable performance status.

## Materials and Methods

### Study patients

We retrospectively reviewed the clinical charts of all patients with histology‐proven malignancies and DM who were referred for radiotherapy (RT) to the Chang Gung Memorial Hospital between January 2000 and December 2012. Patients with hepatocellular carcinoma with a nodular size larger than 1 cm and typical features on at least two dynamic imaging techniques were also eligible 23. The exclusion criteria were as follows: (1) no height or weight measured within 2 weeks before and 1 day after the initiation of RT, (2) Eastern Cooperative Oncology Group (ECOG) performance status (PS) greater than 2 at the time of RT, (3) less than 18 years of age, and (4) lack of information on education and/or employment status. Data collection from electronic medical records was supervised by an experienced nurse and a radiation oncologist.

### Definition of the study variables

We used the World Health Organization's BMI classification system, which includes the following categories: underweight (BMI < 18.5 kg/m^2^), normal‐weight (BMI: 18.5–24.99 kg/m^2^), overweight (BMI: 25–29.99 kg/m^2^), and obese (BMI ≥ 30 kg/m^2^). The time of onset of metastases was defined as the number of months elapsed between the diagnosis of primary cancer and the identification of DM. For the analysis, it was categorized as >12 months and ≤12 months. Multiple metastases were considered to be present when more than two organs or different parts of the skeleton (e.g., sternum and sacrum) were involved simultaneously. The sites of DM were categorized as (1) bone metastases, (2) brain metastases, or (3) metastases at other sites. Primary tumors were classified into one of the following three categories: (1) lung cancer, (2) breast cancer, and (3) other cancers. Because of different radiation doses and fraction sizes, the total equivalent dose in 2 Gy fractions (EQD_2 Gy_) was used for analysis. Systemic treatments (chemotherapy, hormonal therapy, or targeted therapy) were recorded starting from 1 month before RT to the date of the last follow‐up. The history of risky oral habits (betel quid chewing, cigarette smoking, and alcohol drinking) was collected by means of a questionnaire at the date of first consultation with a radiation oncologist. Betel quid chewers were classified as current chewers or former chewers and never‐chewers. Similarly, alcohol drinkers were divided into current drinkers or former drinkers and never‐drinkers. History of cigarette smoking before the diagnosis of cancer was categorized according to the Centers of Disease Control and Prevention classification system as never smoking (subject who have smoked less than 100 cigarettes in their lifetime and who currently do not smoke cigarettes) or current/former smoking (subject who have smoked at least 100 cigarettes in their lifetime) 24. The presence of comorbidities was assessed using the Charlson comorbidity index and dicothomized as yes or no. In this study, the presence of a metastatic solid tumor (corresponding to a score of (6) was categorized as no 25. Information concerning the highest level of education attained was obtained for all patients by an interview at the time of cancer diagnosis. Education level was categorized as low (no education or elementary school) or high (junior school and higher). Place of residence was categorized as urban (patient living in a municipality directly under the Central Government or in an urbanized area having more than eight hundred people per square kilometer) or rural. Employment status was categorized into three levels according to the Social Classes of the British Registrar General^26^ with slight modifications, as follows: no employment, low employment status (manual employees, unskilled or semiskilled workers), and high employment status (nonmanual skilled workers, managers, and professional workers).

### Statistical analysis

OS – defined as the time (in months) from the date of the first RT for DM to the date of death – was the main outcome measure. The Chi‐square test was used to assess differences in clinical parameters across different BMI categories. Survival curves were plotted using the Kaplan–Meier method and compared with the log‐rank test. A multivariable Cox proportional hazards model was used to identify the independent predictors of OS. Results were expressed as hazard ratios (HRs) with their 95% confidence intervals (CIs). All the patient‐, tumor‐, and treatment‐related variables were included as potential predictors in multivariable analysis, the only exceptions being the presence of comorbidities (*P *=* *0.895 in univariate analysis) and place of residence (*P *=* *0.197 in univariate analysis). Two‐tailed *P* values <0.05 were considered statistically significant.

## Results

### General characteristics of the study patients

Between 2000 and 2012, a total of 7345 cancer patients with DM who required radiation treatment were identified. We excluded (1) a total of 2,339 cases with a ECOG PS of 3 or 4, (2) 573 patients with missing information on height and weight,(3) 304 patients who lacked official pathological reports, (4) 47 patients with missing information on educational level and/or employment status, (5) 14 patients who were younger than 18 years of age, and (6) 58 patients with massive ascites. Consequently, the final study sample consisted of 4010 patients. Table 1 summarizes the general characteristics of the study participants. In the entire study cohort, the mean BMI at the time of RT was 22.99 kg/m^2^. Specifically, 380 (9.5%), 2251 (63.6%), 925 (23.1%), and 154 (3.8%) patients were classified as underweight, normal‐weight, overweight, and obese, respectively. The relation between BMI and sites of primary cancer is depicted in supplementary Table 1.

**Table 1:** Baseline characteristics of underweight, normal‐weight, overweight, and obese cancer patients with distant metastases

	BMI (kg/m^2^)				Entire cohort	*P* value
	<18.5	18.5–24.99	25–29.99	≥30		
Number of patients	380 (9.5%)	2551 (63.6%)	925 (23.1%)	154 (3.8%)	4010 (100%)	
Age (years), median	57.0 (19.9–87.7)	59.8 (18.4–94)	60 (24.2–90.3)	57.5 (18.9–90.3)	59.7 (18.4–94)	<0.001^b^
Mean (±SD)	56.99 ± 14.83	59.60 ± 12.72	60.15 ± 12.08	58.53 ± 12.73	59.44 ± 12.81	
Sex						<0.001^a^
Female	177 (46.6%)	1125 (44.1%)	401 (43.4%)	102 (66.2%)	1805 (45.0%)	
Male	203 (53.4%)	1426 (55.9%)	524 (56.6%)	52 (33.8%)	2205 (55.0%)	
Performance status						0.088^a^
ECOG 0/1	263 (69.2%)	1880 (73.7%)	693 (74.9%)	121 (78.6%)	2957 (73.7%)	
ECOG 2	117 (30.8%)	671 (26.3%)	232 (25.1%)	33 (21.4%)	1053 (26.3%)	
Sites of metastasis						<0.001^a^
Bone	193 (50.8%)	1314 (51.5%)	443 (47.9%)	76 (49.4%)	2026 (50.5%)	
Brain	135 (35.5%)	976 (38.3%)	382 (41.3%)	46 (29.9%)	1539 (38.4%)	
Others	52 (13.7%)	261 (10.2%)	100 (10.8%)	32 (20.8%)	445 (11.1%)	
Onset of metastasis						0.022^a^
≤1 year	208 (54.7%)	1510 (59.2%)	508 (54.9%)	78 (50.6%)	2304 (57.5%)	
>1 years	172 (45.3%)	1041 (40.8%)	417 (45.1%)	76 (49.4%)	1706 (42.5%)	
Onset of metastasis (years), median	0.79 (0.0–13.44)	0.67 (0–16.71)	0.79 (0–27.75)	0.97 (0–10.10)	0.72 (0–27.75)	0.290^b^
Mean (±SD)	1.70 ± 2.33	1.53 ± 2.22	1.62 ± 2.43	1.79 ± 2.19	1.58 ± 2.29	
Metastases at more than one site						0.494^a^
No	177 (46.6%)	1125 (44.1%)	425 (45.9%)	75 (48.7%)	1802 (44.9%)	
Yes	203 (53.4%)	1426 (55.9%)	500 (54.1%)	79 (51.3%)	2208 (55.1%)	
Site of primary cancer						0.022^a^
Lung	146 (38.4%)	1091 (42.8%)	374 (40.4%)	49 (31.8%)	1660 (41.4%)	
Breast	35 (9.2%)	268 (10.5%)	111 (12.0%)	25 (16.2%)	439 (10.9%)	
Other sites	199 (52.4%)	1192 (46.7%)	440 (47.6%)	80 (51.9%)	1911 (47.7%)	
EQD_2 Gy_						<0.000^b^
Median (Gy)	32.5 (2.0–60.6)	32.5 (1.4–84.0)	32.5 (3.3–104.0)	32.5 (0.9–83.3)	32.5 (0.9–104.0)	
EQD_2 Gy_						<0.001^a^
<32 Gy	183 (48.2%)	1142 (44.8%)	340 (36.8%)	63 (40.9%)	1728 (43.1%)	
≥32 Gy	197 (51.8%)	1409 (55.2%)	585 (63.2%)	91 (59.1%)	2282 (56.9%)	
Systemic therapy						<0.001^a^
No	235 (61.8%)	1294 (50.7%)	421 (45.5%)	57 (37.0%)	2007 (50.0%)	
Yes	145 (38.2%)	1257 (49.3%)	504 (54.5%)	97 (63.0%)	2003 (50.0%)	
Comorbidities						<0.001^a^
No	301 (79.2%)	1792 (70.2%)	567 (61.3%)	76 (49.4%)	2736 (68.2%)	
Yes	79 (20.8%)	759 (29.8%)	358 (38.7%)	78 (50.6%)	1274 (31.8%)	
Employment status						0.002^a^
High	85 (22.4%)	525 (20.6%)	216 (23.4%)	30 (19.5%)	856 (21.3%)	
Low	101 (26.6%)	865 (33.9%)	286 (30.9%)	34 (22.1%)	1286 (32.1%)	
None	194 (51.1%)	1161 (45.5%)	423 (45.7%)	90 (58.4%)	1868 (46.6%)	
Education level						<0.001^a^
None/primary school	169 (44.5%)	1362 (53.4%)	546 (59.0%)	94 (61.0%)	2171 (54.1%)	
High school	211 (55.5%)	1189 (46.6%)	379 (41.0%)	60 (39.0%)	1839 (45.9%)	
Place of residence						0.440^a^
Urban	226 (59.5%)	1418 (55.6%)	525 (56.8%)	82 (53.2%)	2251 (56.1%)	
Rural	154 (40.5%)	1133 (44.4%)	400 (43.2%)	72 (46.8%)	1759 (43.9%)	
Cigarette smoking						<0.001^a^
No	202 (53.2%)	1469 (57.6%)	545 (58.9%)	115 (74.7%)	2331 (58.1%)	
Yes	178 (46.8%)	1082 (42.4%)	380 (41.1%)	39 (25.3%)	1679 (41.9%)	
Betel quid chewing						0.693^a^
No	324 (85.3%)	2188 (85.8%)	798 (86.3%)	137 (89.0%)	3447 (86.0%)	
Yes	56 (14.7%)	363 (14.2%)	127 (13.7%)	17 (11.0%)	563 (14.0%)	
Alcohol drinking						0.009^a^
No	261 (68.7%)	1834 (71.9%)	661 (71.5%)	128 (83.1%)	2884 (71.9%)	
Yes	119 (31.3%)	717 (28.1%)	264 (28.5%)	26 (16.9%)	1126 (28.1%)	
Overall survival (months), median	3.23 (0.13–122.17)	6.08 (0.03–149.46)	7.99 (0.07–165.21)	12.49 (0.19–48.11)	6.35 (0.33–165.21)	<0.001^b^
Mean (±SD)	8.09 ± 13.17	11.75 ± 16.16	15.21 ± 21.94	20.97 ± 6.39	12.56 ± 18.07	

BMI, body mass index; SD, standard deviation; ECOG, Eastern Cooperative Oncology Group; EQD_2 Gy_, equivalent dose in 2 Gy fractions.

a Two‐tailed chi‐square test.

b Two‐tailed analysis of variance (ANOVA) test.

The median age of the study patients was 59.6 years (range: 18.4–94 years). A total of 2,245 patients had their height and weight measured on the day of radiation. There were 2957 (73.7%) patients who had an ECOG PS of 0 or 1. The median time between the diagnosis of primary cancer and the detection of DM was 8.64 months (range: 0–27.75 years). The median time from development of DM to time to referral for the first course of radiation was 0.4 months (range: 0–93 months). A total of 296 (7.4%) patients remained DM‐free for more than 5 years after the initial diagnosis of primary cancer. Of the 4010 patients, 1660 (41.4%) had lung cancer, 439 (10.6%) breast cancer, and 1911 (47.6%) other types of cancer. Bone was the most common site of DM in patients requiring RT. Supplementary Table 2 summarizes the relation between the sites of DM and primary cancer sites. The median EDQ_2 Gy_ was 32.5 Gy (range: 0.92–104 Gy).

**Table 2:** Univariate and multivariable analysis of factors predicting overall survival in cancer patients with distant metastases

Total number of patients = 4010	Univariate analysis		Multivariable analysis	
	Overall survival		Overall survival	
	HR (95% CI)	*P* value	HR (95% CI)	*P* value
Body mass index				
Underweight vs. normal‐weight	1.421 (1.273–1.586)	<0.001	1.410 (1.261–1.577)	<0.001
Overweight vs. normal‐weight	0.825 (0.763–0.893)	<0.001	0.840 (0.776–0.909)	<0.001
Obese vs. normal‐weight	0.596 (0.499–0.712)	<0.001	0.676 (0.565–0.809)	<0.001
Age (≥59.75 years vs. <59.75 years)	1.204 (1.129–1.284)	<0.001	1.050 (0.971–1.135)	0.224
Sex (male vs. female)	1.517 (1.422–1.620)	<0.001	1.305 (1.182–1.441)	<0.001
Performance status (ECOG 2 vs. 0/1)	1.111 (1.034–1.195)	0.004	1.134 (1.054–1.220)	0.001
Site of metastasis				
Brain vs. bone	1.024 (0.957–1.097)	0.490	1.053 (0.974–1.138)	0.191
Other sites vs. bone	0.840 (0.754–0.935)	0.001	1.133 (1.006–1.277)	0.040
Onset (>1 years vs. ≤1 year)	0.801 (0.751–0.855)	<0.001	0.910 (0.847–0.977)	0.009
Multiple metastases (yes vs. no)	1.191 (1.116–1.271)	<0.001	1.180 (1.100–1.267)	<0.001
Site of primary cancer				
Breast vs. lung	0.593 (0.530–0.664)	<0.001	0.810 (0.712–0.923)	0.002
Other sites vs. lung	0.840 (0.754–0.935)		0.850 (0.785–0.921)	<0.001
EQD_2 Gy_ (≥32 Gy vs. <32 Gy)	0.745 (0.698–0.794)	<0.001	0.803 (0.752‐0.858)	<0.001
Chemotherapy (yes vs. no)	0.657 (0.616–0.700)	<0.001	0.713 (0.667–0.763)	<0.001
Comorbidities (yes vs. no)	0.995 (0.929–1.066)		1.011 (0.940–1.087)	0.767
Employment status				
Low vs. high	1.125 (1.029–1.231)	0.010	0.998 (0.908–1.097)	0.963
None vs. high	1.027 (0.944–1.117)	0.538	1.106 (1.007–1.216)	0.036
Education level (high vs. low)	0.867 (0.813–0.925)	<0.001	0.912 (0.845–0.984)	0.017
Place of residence (urban vs. rural)	1.043 (0.978–1.113)	0.197	1.004 (0.940–1.072)	0.902
Cigarette smoking (yes vs. no)	1.454 (1.362–1.552)	<0.001	1.100 (0.998–1.211)	0.054
Betel quid chewing (yes vs. no)	1.438 (1.313–1.575)	<0.001	1.202 (1.081–1.337)	0.001
Alcohol drinking (yes vs. no)	1.320 (1.230–1.417)	<0.001	1.007 (0.921–1.101)	0.880

HR, hazard ratio; CI, confidence interval; BMI, body mass index; ECOG, Eastern Cooperative Oncology Group; EQD_2 Gy_, equivalent dose in 2 Gy fractions.

### Overall survival and its predictors

**Figure 1:**
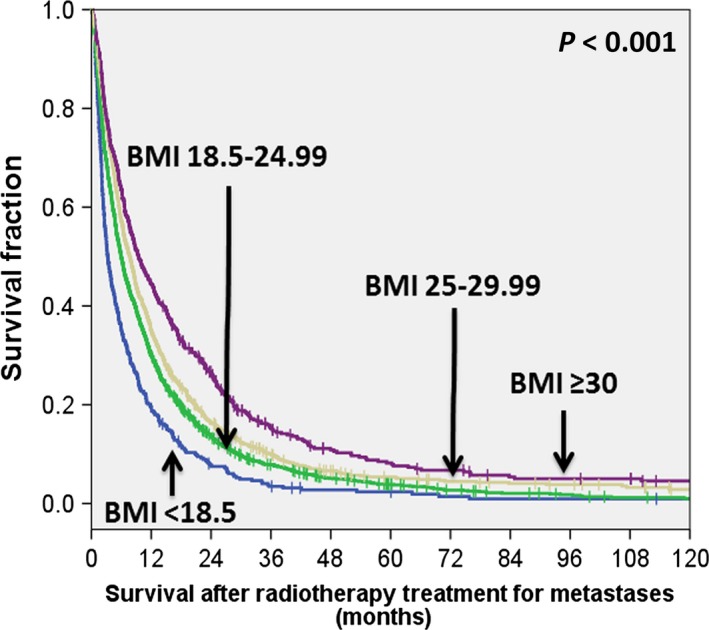
Kaplan–Meier overall survival plots of underweight, normal‐weight, overweight, and obese cancer patients with distant metastases.

The study patients were followed up for a minimum of 12 months or until death. The median follow‐up time for surviving patients was 24.43 months (range: 0.13–164.1 months). A total of 3683 deaths occurred during the follow‐up period. The 12‐ and 24‐month OS rates for the entire study cohort were 32.3% and 15.5%, respectively. The median OS was 6.35 months, with a range from 1 day to 165 months. Table 1 shows the median and mean OS of patients according to their BMI values. Figure 1 depicts the OS according to their BMI values. Patients with high BMI had better OS rates than those with low BMI values. Specifically, the median OS time was 3.23 months (range: 0.1–122.17 months) for underweight patients, 6.08 months (range: 0.03–149.46 months) for normal‐weight patients, 7.99 months (range: 0.07–158.01 months) for overweight patients, and 12.49 months (range, 0.2–164.1 months) for obese patients (log‐rank test: *P *<* *0.001). Besides BMI, the following variables were identified as significant adverse predictors of OS in univariate analyses: male sex, older age, skeletal metastases, multiple metastases, poor ECOG PS, metastases occurring more than 1 year after the initial diagnosis of cancer, primary gastrointestinal malignancies, low EDQ_2 Gy_, lack of systemic treatment, low occupational level, lower educational level, current smoking, betel quid chewing, and alcohol drinking (Table 2). After allowance for potential confounders, BMI retained its independent prognostic significance for OS (Fig. 1). Compared with normal‐weight patients, both obese (HR = 0.676; 95% CI = 0.565–0.809, *P *<* *0.001) and overweight individuals (HR = 0.84; 95% CI = 0.776–0.909, *P *<* *0.001) had a reduced risk of all‐cause mortality. Conversely, underweight patients had a significantly higher risk of death from all causes (HR = 1.41; 95% CI = 1.261–1.577, *P *<* *0.001; Table 2. The adverse prognostic significance of low BMI was confirmed in all the following patient subgroups: male subjects, patients with poor PS, patients with short time intervals between primary cancer diagnosis and onset of DM, subjects having more than one metastatic site, patients with primary malignancies other than lung cancer, subjects who received low radiation doses to metastastic sites, patients who did not receive systemic treatment, unemployed subjects, and patients with a history of betel quid chewing (Table 2).

**Figure 2:**
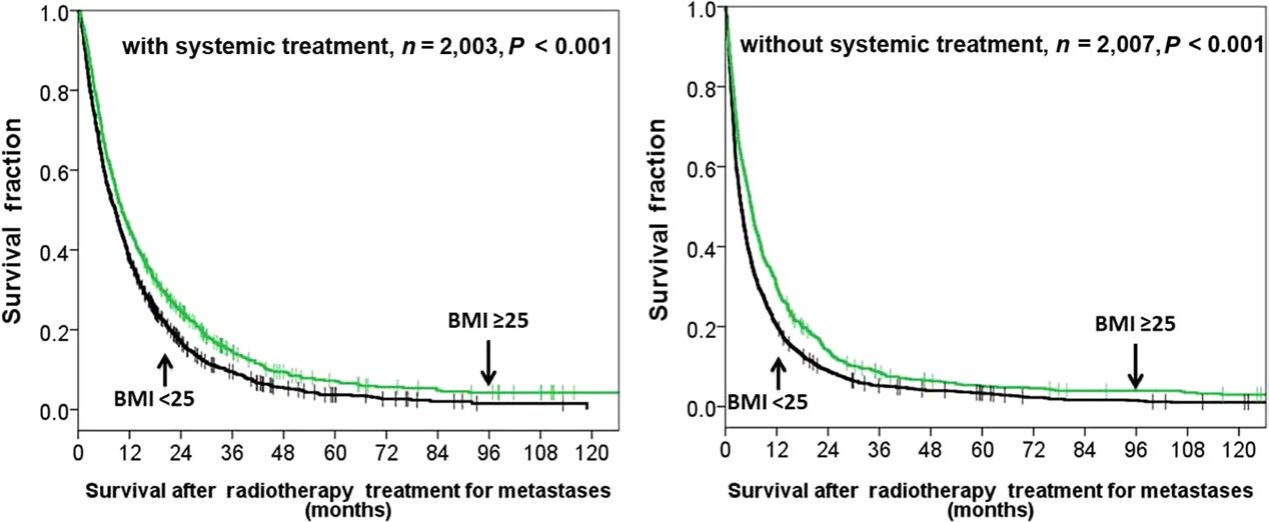
Overall survival according to high BMI (≥25 kg/m^2^) versus lower BM (<25 kg/m^2^) in patients with systemic treatment and without systemic treatment.

**Figure 3:**
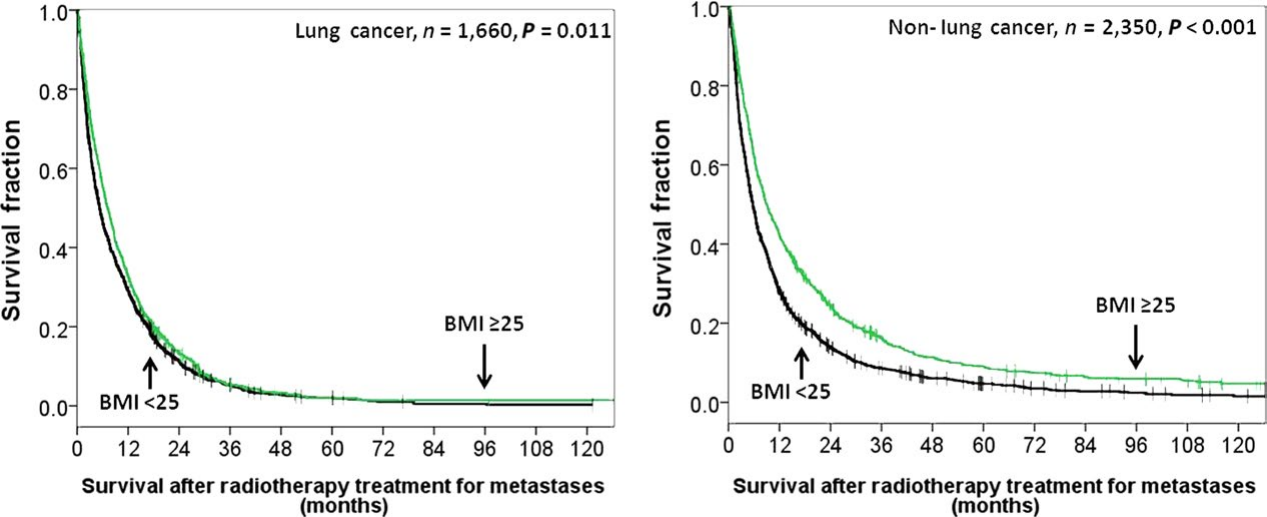
Overall survival according to high BMI (≥25 kg/m^2^) versus lower BM (<25 kg/m^2^) in patients with primary lung cancer and nonlung cancer.

An analysis of the subgroup of patients who received systemic treatment or not, we found a statistically significant OS benefit with higher BMI (BMI > /25 kg/m^2^) in patients with systemic treatment (HR 0.797, 95% CI 0.726–0.876%, *P* < 0.001) and a similar survival benefit observed in patients without systemic treatment (HR 0.759, 95% CI 0.69–0.835, *P* < 0.001) (Fig. 2). In the subgroup analysis of primary lung cancer and others, we found a statistically significant OS benefit with higher BMI (BMI > /25 kg/m^2^) in patients with primary lung cancer (HR 0.875, 95% CI 0.79–0.97%, *P* = 0.011) and in patients with other primary cancer types (HR 0.707 95% CI 0.647–0.771, *P* < 0.001) (Fig. 3).

## Discussion

The results of this retrospective study conducted in 4010 cancer patients with DM and an ECOG PS of 0–2 demonstrate that overweight and obesity are favorable prognostic factors for OS even after allowance for potential confounders. Accordingly, both obese (HR = 0.676) and overweight patients (HR = 0.840) had a lower risk of all‐cause mortality when compared with normal‐weight patients. Conversely, underweight was an adverse prognostic factor and was associated with a higher risk of death. To our knowledge, this is the largest study to date to analyze the prognostic significance of BMI in cancer patients with DM. Our current results are in accordance with previous data showing a favorable prognostic significance of overweight and obesity in different cohorts of patients with chronic diseases, including rheumatoid arthritis, cardiovascular disease, chronic obstructive pulmonary disease, and chronic renal failure 9, 10, 27. Although BMI is frequently considered as a surrogate index for assessing excess body fat, it could also serve as a proxy for the global nutritional status 28. The prognostic value of BMI has been extensively studied in DM‐free cancer patients, but its exact significance in metastatic patients remains unclear. It is well‐known that the presence of DM is frequently associated with significant weight loss, skeletal muscle wasting, and loss of adipose tissue. Because massive ascites may increase a patient's body weight without representing a form of overweight or obesity, we carefully excluded such subjects from this study. Skeletal muscle wasting, termed sarcopenia, is a common feature of aging and can be frequently found in cancer patients with DM. Previous studies have consistently shown that sarcopenia has an adverse prognostic significance in patients with malignancies, being associated with increased treatment toxicity and higher mortality rates 29, 30, 31. Sarcopenia – which is invariable associated with reduced BMI values – is generally considered as a manifestation of chronic inflammation. In this regard 31, 32, increased levels of proinflammatory interleukin 6 (IL‐6) have been associated with sarcopenia, poor PS, and low BMI 33, 34. Lederle et al. have also shown that IL‐6 can orchestrate a complex activation of proinflammatory and angiogenic factors that may ultimately drive and promote tumorigenesis both in vitro and in vivo 35. Moreover, IL‐6 has been shown to trigger epithelial‐mesenchymal transformation in breast cancer cells, potentially promoting DM 36. Notably, the use of IL‐6 receptor antagonists or monoclonal antibodies directed against IL‐6 has been shown to dramatically inhibit the development of cancer‐associated muscle wasting and cachexia. 37, 38.

Another important cause of cancer cachexia is adipose tissue loss. Although BMI is generally considered as a poor proxy for body composition and the presence of adipose tissue 39, it has been shown to be positively correlated with waist circumference and negatively associated with mortality 40. Importantly, BMI has an inverse association with fatty acid synthase (*FASN*) expression. Besides being involved in fatty acid synthesis, *FANS* is an oncogene which has been found to be overexpressed in several malignancies. Hakimi et al. 41. have reported that *FANS* is significantly downregulated in obese patients with renal cell carcinoma and has favorable effects on cancer‐specific survival, thus serving as a potential molecular effector of the so‐called “obesity paradox”. We are currently conducting a study on the associations between *FANS* expression, fatty acid levels, and survival in patients with metastatic head‐and‐neck cancer. Previous studies focusing on the alterations of body composition in patients with malignancies yielded conflicting results. Some reports indicated a predominant loss of body fat, whereas others pointed to a primary role of sarcopenia 30, 42, 43. Such discrepancies may be at least in part explained by distinct techniques used to assess body composition and different baseline characteristics of the clinical cohorts. Currently, dual‐energy X‐ray absorptiometry (DEXA), CT, and MRI are the most commonly used techniques for body composition assessment. Although the use of DEXA results in a lower radiation exposure 44, CT scans allow a precise discrimination between muscle and adipose tissue. Because CT imaging is routinely performed for disease staging and treatment monitoring in patients with malignancies, its use for determining body composition may be highly convenient and cost‐effective. Another point the merits consideration when dealing with the prognostic significance of BMI in cancer patients is the time of its determination. Differently from lean body mass, reductions in adipose tissue may become more pronounced in advanced disease stages. In patients with metastastic lung and gastrointestinal cancers, Murphy et al. have reported that the loss of adipose tissue markedly accelerates at 7 months before death. Notably 42, Fouladiunet and colleagues have reported that whole‐body adipose tissue mass and daily fat intake are better predictors of survival than lean mass and protein intake in cancer patients with DM undergoing palliative treatment 43.

Tumors of high malignant potential are supposed to require higher levels of energy for growing. Elevated adipose tissue lipolysis and increasing fatty acid oxidation is essential to provide energy. We attribute greater fat loss in metastatic patients to aggressive tumor behavior with higher energy demand. In our unpublished data, we found BMI is strongly correlated with percent body fat but not with percent body muscle in patients with head and neck cancer. It may explain the lower BMI in metastatic patients reflects higher malignant potential which is associated with poorer overall survival.

Some caveats of our study deserve consideration. First, BMI values were collected when patients received their first RT for the presence of DM. Because of the long follow‐up period 10, the retrospective collection of serial BMI changes may pose significant challenges. The majority of the study participants did not undergo BMI measurements at the time of DM diagnosis and regular BMI assessments were not planned. Another limitation is that body composition measures were not available for this study.

Noteworthy, it is possible that there was a selection bias in this retrospective study because systemic treatment was likely delivered to patients with higher BMI leading to longer survival than those with lower BMI. In our study, systemic treatment was delivered in 63% of patients with BMI > 30 kg/m^2^ versus 38.2% of patients with BMI < 18.5 kg/m^2^. However, after subgroup analysis, we found higher BMI (BMI ≥ 25 kg/m^2)^ results in survival benefits regardless whether patients received systemic treatment (HR 0.797 and HR 0.759). Regarding the effect of BMI on patients with primary lung cancer or nonlung cancer, we showed metastatic patients with higher BMI had longer OS in both groups (HR 0.875 and HR 0.707).

The median time from development of DM to time to referral for the first course of radiation (it is the time for BMI measurement) was 0.4 months. Because of the short interval between development of DM and BMI measurement, it is reasonably inferred that BMI at the time of referral for first of course of radiation can represent the patients' condition with development of DM. Since our hospital is a tertiary referral center, BMI data were not available in some patients at the time of development of DM diagnosed at primary or secondary hospital. In our radiation department, BMI measurement is routine for every consulting patients with ECOG 0 ~ 2. Therefore, we think BMI measurement at this time point is a reliable prognosticator of OS in metastatic patients.

In conclusion, our findings indicate that overweight and obesity are independent predictors of better OS rates in cancer patients with DM and a good performance status. The question as to whether the favorable prognostic significance of high BMI values is mediated by inflammatory mediators, altered expression of genes regulating fat metabolism 41, or other factors remains open. Accurate measurements of body composition and additional molecular studies may further disentangle the mechanisms behind the prognostic value of BMI observed in our cohort. Notwithstanding its caveats, this study may have clinical implications for the clinical management of patients with DM and a favorable performance status. Specifically, our results may stimulate further research aimed at assessing whether aggressive treatments for maintaining body weight may improve clinical outcomes in metastatic patients.

## Conflict of Interest

The authors declare that they have no conflict of interest.

## Supporting information


**Table S1.** Primary cancer sites in underweight, normal‐weight, overweight, and obese cancer patients with distant metastases.

**Table S2.** Sites of distant metastases in relation to primary cancer sites.
