# Supplementary material for: Overweight and obesity predict better overall survival rates in cancer patients with distant metastases
Source: Cancer Med. 2016 Jan 26;5(4):665–75. doi: 10.1002/cam4.634 (PMC4831285; doi:10.1002/cam4.634)
Supplement: Supplementary file 1 — Table S1. Primary cancer sites in underweight, normal‐weight, overweight, and obese cancer patients with distant metastases. [file CAM4-5-665-s001.docx]

| Supplementary table 1. Primary cancer sites in underweight, normal-weight, overweight, and obese cancer patients with distant metastases | | | | | | | |
| --- | --- | --- | --- | --- | --- | --- | --- |
|  | BMI (kg/m^2^) | | | | Entire cohort | | P value |
|  | <18.5 | 18.5-24.9 | 25-29.9 | <30 | |  |  |
| Number of patients  (%) | 380  (9.5%) | 2551  (63.6%) | 925  (23.1%) | 154  (3.8%) | | 4010 |  |
| Primary cancer site |  |  |  |  | |  | <0.001^a^ |
| **Lung** | **146 (38.4%)** | **1094 (42.8%)** | **374 (40.4%)** | **49 (31.8%)** | | **1660 (41.4%)** |  |
| **Non-lung** | **234 (61.6%)** | **1,457 (57.2%)** | **551 (59.6%)** | **105 (68.2%)** | | **2,350 (58.6%)** |  |
| Gastrointestinal | 55 (14.5%) | 315 (12.3%) | 93 (10.1%) | 11 (7.1%) | | 474 (11.8%) |  |
| Breast | 35 (9.2%) | 268 (10.5%) | 111 (12.0%) | 25 (16.2%) | | 439 (10.9%) |  |
| Colorectal | 33 (8.7%) | 227 (8.9%) | 80 (8.6%) | 13 (8.4%) | | 353 (8.8%) |  |
| Urinary tract | 13 (3.4%) | 160 (6.3%) | 96 (10.4%) | 10 (6.5%) | | 279 (7%) |  |
| Gynecological | 25 (6.6%) | 123 (4.8%) | 54 (5.8%) | 28 (18.2%) | | 230 (5.7%) |  |
| Unknown | 16 (4.2%) | 125 (4.9%) | 49 (5.3%) | 7 (4.5%) | | 197 (4.9%) |  |
| Oral cavity | 18 (4.7%) | 81 (3.2%) | 26 (2.8%) | 5 (3.2%) | | 130 (3.2%) |  |
| Nasopharyngeal | 20 (5.3%) | 75 (2.9%) | 20 (2.2%) | 1 (0.6%) | | 116 (2.9%) |  |
| Pharyngolaryngeal | 12 (3.2%) | 32 (1.3%) | 6 (0.6%) | 1 (0.6%) | | 51 (1.3%) |  |
| Skin | 2 (0.5%) | 29 (1.1%) | 13 (1.4%) | 3 (1.9%) | | 47 (1.2%) |  |
| Sarcoma | 5 (1.3%) | 25 (1.0%) | 3 (0.3%) | 1 (0.6%) | | 34 (0.8%) |  |
| ^a^Two-tailed χ^2^ test. | | | | | | | |
