# Supplementary material for: Overweight and obesity predict better overall survival rates in cancer patients with distant metastases
Source: Cancer Med. 2016 Jan 26;5(4):665–75. doi: 10.1002/cam4.634 (PMC4831285; doi:10.1002/cam4.634)
Supplement: Supplementary file 2 — Table S2. Sites of distant metastases in relation to primary cancer sites. [file CAM4-5-665-s002.docx]

| Supplementary table 2. Sites of distant metastases in relation to primary cancer sites | | | | | |
| --- | --- | --- | --- | --- | --- |
|  | Bone metastasis | Brain metastasis | Metastasis at  other sites | Entire cohort | P value |
| Number of patients  (%) | 2026 (50.5%) | 1539  (38.4%) | 445  (11.1%) | 4010 |  |
| Primary cancer site |  |  |  |  | <0.001^a^ |
| Lung | 671 (33.1%) | 957 (62.2%) | 32 (7.2%) | 1660 (41.4%) |  |
| Non-lung | 1355 (66.9%) | 582 (37.8%) | 413 (92.8%) | 2350 (58.6%) |  |
| Gastrointestinal | 338 (16.7%) | 66 (4.3%) | 70 (15.7%) | 474 (11.8%) |  |
| Breast | 193 (9.5%) | 231 (15.0%) | 15 (3.4%) | 439 (10.9%) |  |
| Colorectal | 200 (9.9%) | 71 (4.6%) | 82 (18.4%) | 353 (8.8%) |  |
| Urinary tract | 219 (10.8%) | 34 (2.2%) | 26 (5.8%) | 279 (7.0%) |  |
| Gynecological | 55 (2.7%) | 33 (2.1%) | 142 (31.9%) | 230 (5.7%) |  |
| Unknown | 103 (5.1%) | 93 (6.0%) | 1 (0.2%) | 197 (4.9%) |  |
| Oral cavity | 78 (3.8%) | 16 (1.0%) | 36 (8.1%) | 130 (3.2%) |  |
| Nasopharyngeal | 101 (5%) | 0 (0.0%) | 15 (3.4%) | 116 (2.9%) |  |
| Pharyngolaryngeal | 32 (1.6%) | 6 (0.4%) | 13 (2.9%) | 51 (1.3%) |  |
| Skin | 21 (1.0%) | 21 (1.4%) | 5 (1.1%) | 47 (1.2%) |  |
| Sarcoma | 15 (0.7%) | 11 (0.7%) | 8 (1.8%) | 34 (0.8%) |  |
| ^a^Two-tailed χ^2^ test. | | | | | |
